# Awareness Among Healthcare Professionals Regarding Contaminated Stethoscopes as a Source of Nosocomial Infections

**DOI:** 10.7759/cureus.5968

**Published:** 2019-10-22

**Authors:** Desaar Zehra, Mishal Iqbal, Ayesha Safdar, Hamza Jamil, Syed Hashim Ali Inam, Muhammad A Zahid

**Affiliations:** 1 Internal Medicine, Army Medical College, Rawalpindi, PAK; 2 Internal Medicine, Military Hospital, Rawalpindi, PAK; 3 Epidemiology and Public Health, Army Medical College, Rawalpindi, PAK; 4 General Medicine, Army Medical College, Rawalpindi, PAK

**Keywords:** nosocomial infections, awareness, stethoscope, pakistan

## Abstract

Objectives

The objective of this study was to determine the awareness among healthcare professionals regarding stethoscopes as a source of nosocomial infections, their cleaning practices in this regard before or after examination, and to find out about the existence of any guidelines and accountability criteria issued by the hospitals in this regard.

Methodology

A descriptive cross-sectional study of 243 healthcare professionals using non-probability convenience sampling was done to include consultants, residents, final-year medical students, and nursing staff and excluding medical students from pre-clinical years as well as doctors of those departments with infrequent use of a stethoscope. The study was conducted for a period of nine months at tertiary health care facilities of Rawalpindi and Islamabad. A self-administered structured questionnaire was used for data collection.

Results

Participants from both genders included 54 participants (22.2%) from the final year, 48 (19.8%) house officers, 106 (43.6%) postgraduate trainees, nine (3.7%) specialists, and 26 (10.7%) nurses. A total of 210 (86.4%) were aware of stethoscopes as a source of nosocomial infections. Among participants, 23 (9.5%) cleaned their stethoscope per patient, 50 (20.6%) did it daily, 48 (19.8%) did it weekly, 41 (16.9%) did it monthly, 12 (4.9%) participants cleaned it six-monthly while 69 (28.4%) respondents had never cleaned their stethoscope. Almost 127 participants (52.3%) used alcohol wipes to clean their stethoscopes, 11 (4.5%) used a wet cloth, six (2.5%) used tissue paper. Sixty-one (24.9%) agreed that the hospital issued protocols for the decontamination of stethoscopes while 189 (77.8%) did not. A total of 241 (99.2%) believed that there were no accountability criteria set for the assessment of the cleanliness of stethoscopes in their hospitals.

Conclusion

A majority of the participants were aware of stethoscopes being a source of nosocomial infections and believed in cleaning stethoscopes regularly. However, a majority of the participants believed that their hospital did not issue any protocols for the decontamination of stethoscopes. Further research can expand our recommendations.

## Introduction

Nosocomial infections are simply defined as infections acquired in the hospital setting [1]. Hospital sites are a source of infections, and contaminated medical equipment like stethoscopes are home to a wide range of pathogens like Klebsiella, Enterococcus, and Methicillin-resistant Staphylococcus Aureus (MRSA) [2-3].

The increased dependence of present-day doctors on numerous medical instruments for diagnosis has not only helped greatly in assuring early diagnosis and prompt treatment but it also has put the patients at a greater risk for acquiring infections in a hospital. Not only hospital utensils but even the sleeves of lab coats, tables, ornaments, and wristwatches have been known to be a contributing source of nosocomial infections. Among these, one of the prime contributors is known to be a stethoscope [3-4]. It was discovered that the diaphragm of the stethoscope becomes colonized with bacteria quickly - acquiring more pathogens than any part of the doctor’s hand except the fingertips [5].

Many international studies conducted in this regard have revealed alarming rates of bacterial contamination of stethoscopes [6-9]. For instance, a study conducted in India showed that only three of 66 (4.5%) of healthcare providers disinfected their stethoscope every day and none cleaned it per patient [10]. When medical students of the University of Belgrade, Serbia, were surveyed regarding stethoscope hygiene, 79.8% fourth-year students and 81.9% sixth-year students disinfected their stethoscope but, even then, the diaphragm was mostly focused on while the rubber tubing was usually ignored [11].

All agents, i.e. soap and water, 70% isopropyl alcohol, and hypochlorous acid in dilute form proved to be effective in reducing the count of bacteria found on the stethoscopes prior to treating them with these agents [12]. Various studies have found that disinfecting stethoscopes with isopropyl alcohol could eliminate 99% of bacteria [13]. According to the Broome Hospital Infection Control and Regional Infection Control Advisory Group, “The Gold Standard is to clean stethoscopes after contact with each patient using antibacterial wipes (70% isopropyl) alongside mobile phones, pagers and hand washing" [14].

The expenditures of hospital-acquired (nosocomial) infections are massive. It is projected that these infections affect 1.7 million patients, costing approximately $28-33 billion and causing up to 99,000 deaths in U.S. healthcare setups yearly [15]. But in a developing country like Pakistan, the economic burden is much more worrisome and the rate of hospital-acquired infections are as high as 39%, much higher than in the developed world [16].

The objective of this study is to find awareness among health care professionals about the role of the stethoscope in nosocomial infections. This study will also assess if health workers are aware of the risk of nosocomial infections caused by stethoscopes and are taking appropriate measures to prevent them as well as inquire about hospital-issued guidelines and protocols.

## Materials and methods

**Figure 1:**
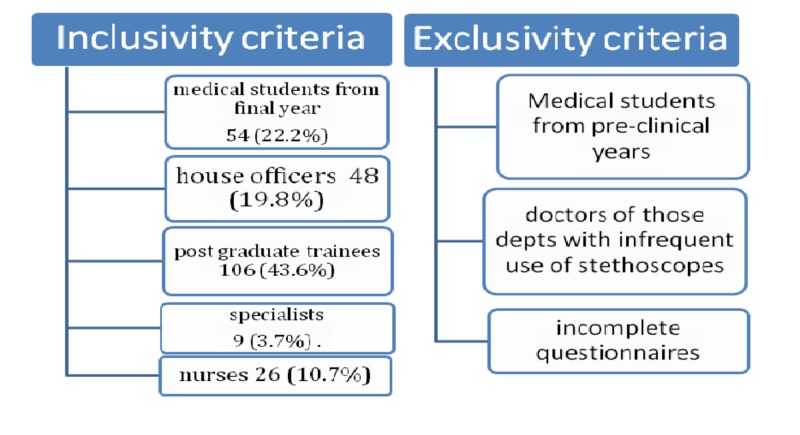
Sampling criteria for the study

A descriptive cross-sectional study was conducted at tertiary government and private hospitals of Rawalpindi and Islamabad from January 2018 to November 2018 after taking ethical approval from the institutional review board of Army Medical College. The non-probability sampling technique was applied. Sample size included 243 healthcare professionals, which included doctors, residents, final-year medical students in their clinical settings, and nursing staff in different departments requiring the frequent use of stethoscopes, after taking informed consent. Medical students from pre-clinical years and doctors of those departments with infrequent use of stethoscopes were excluded. Incomplete questionnaires were discarded at the end of the study (Figure 1).

A well-designed, pre-tested structured questionnaire having both open and close-ended questions was used to collect the data, which included questions to test the awareness of the participants regarding this topic, their source of knowledge, their own cleaning habits as well as inquire about any protocols and accountability measures taken by the hospitals.

## Results

**Figure 2:**
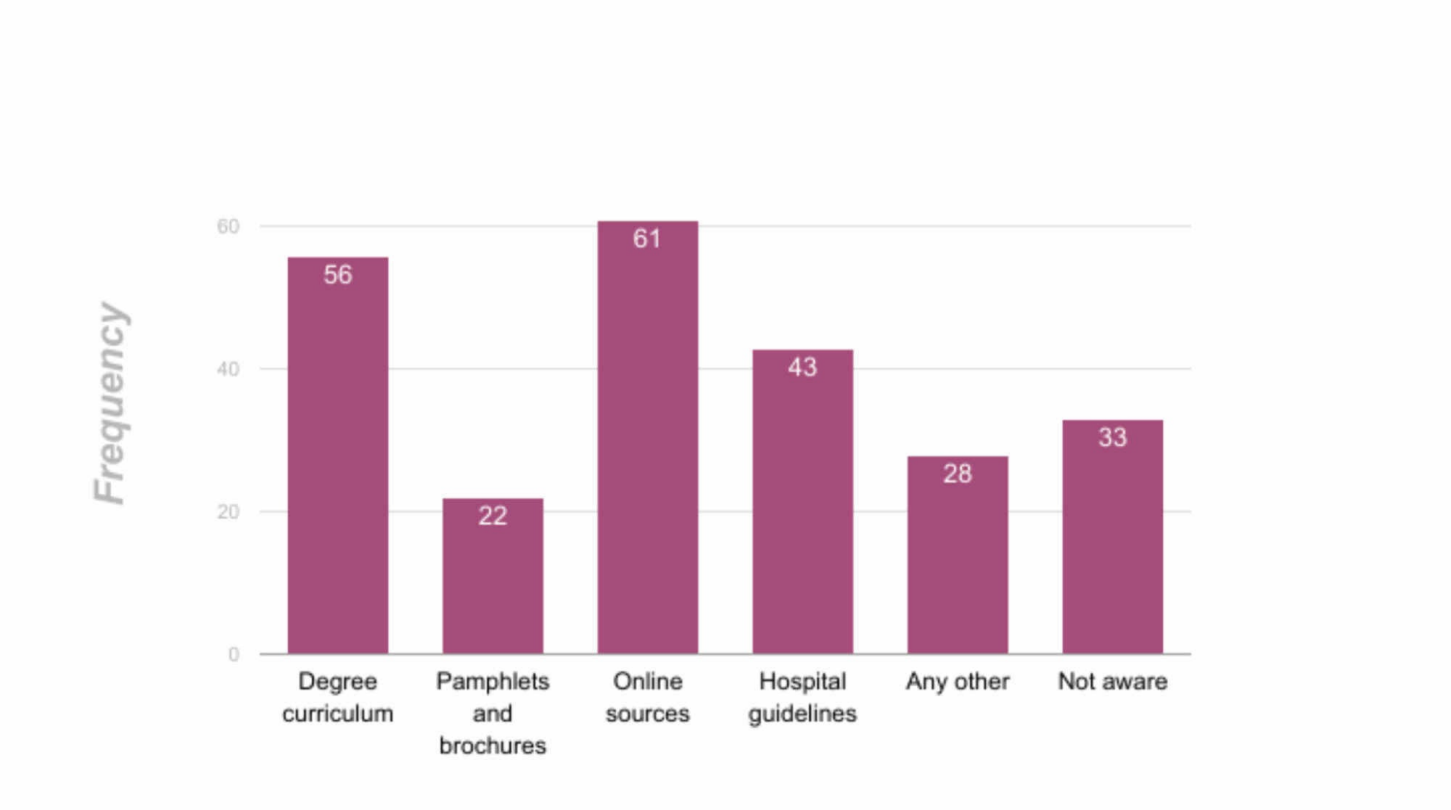
Source of knowledge among participants about the stethoscope as a source of nosocomial infections

Before the commencement of the study, we hypothesized that while most healthcare professionals acknowledge stethoscopes as a source of nosocomial infections, they are not aware of the recommended cleaning practices and that a majority of the hospitals have not issued any standard protocols as well as accountability measures regarding this issue. The hypothesis was tested and proved to hold true.

in the actual study settings, out of the total 243 total participants, including both males and females, there were 54 participants (22.2%) from the final year, 48 (19.8%) house officers, 106 (43.6%) post-graduate trainees, nine (3.7%) specialists, and 26 (10.7%) nurses. Out of a total of 243 participants, 237 (97.5%) had knowledge of nosocomial infections. Out of these 237 participants, 56 (23%) came to know about it as part of their degree curriculum, 22 (9.1%) through posters and pamphlets, 61 (25.5%) through online sources, 43 (17.1%) through hospital guidelines, and 28 (11.5%) through other sources. This is illustrated in Figure 2.

A total of 210 participants (86.4%) were aware of stethoscopes being a source of nosocomial infections. This was in accordance with our hypothesis.

Among the participants, 23 (9.5%) cleaned their stethoscope per patient, 50 (20.6%) did it daily, 48 (19.8%) did it weekly, 41 (16.9%) did it monthly, 12 (4.9%) six-monthly while 69 (28.4%) of the respondents had not ever cleaned their stethoscope. This is illustrated in Table 1.

**Table 1:** Frequency of cleaning stethoscope by the participants

	Frequency	Percent	Valid Percent	Cumulative Percent
Per Patient	23	9.5	9.5	9.5
Daily	50	20.6	20.6	30.0
Weekly	48	19.8	19.8	49.8
Monthly	41	16.9	16.9	66.7
6 Monthly	12	4.9	4.9	71.6
Never Done	69	28.4	28.4	100
Total	243	100	100	

A total of 127 participants (52.3%) used alcohol wipes to clean their stethoscopes while seven (2.9%) used Tuffie wipes, 11 (4.5%) used a wet cloth, six (2.5%) used tissue paper, and 23 (9.5%) used other agents, with Sterillium being the most popular choice.

When asked about the reason for cleaning the stethoscope, high-risk Infections was the most popular reason (30.9%) for making healthcare professional clean their stethoscopes.

On inquiring about the part of the stethoscope being cleaned, only 30 (12.3%) participants cleaned all three parts of the stethoscope, which is the recommended practice. 116 participants (47.7%) cleaned only the diaphragm and 20 (8.23%) cleaned only the earplugs.

Fifty-four participants (22.2%) agreed that the hospital they worked at issue protocols for the decontamination of stethoscopes while 189 (77.8%) did not. Out of these, 42 (17.3%) thought that these protocols were sufficient for preventing the spread of nosocomial infections through stethoscopes while 12 (4.9%) thought otherwise. Two-hundred forty-one (99.2%) believed that there were no accountability criteria set for the assessment of the cleanliness of stethoscopes in their hospitals.

## Discussion

The majority of the participants in our study, ranging from final-year medical students to specialist doctors and nurses, were aware of stethoscopes being a source of nosocomial infections. The majority of the participants also believed in cleaning stethoscopes regularly, yet only 9.5% claimed that they cleaned their stethoscope after every use. Alcohol wipes were the most popular cleaning agent.

It is interesting to see that the percentage of aware participants was recorded higher in our study than in a study conducted in Boston, which reported an awareness percentage of 76%, yet 24% reported disinfecting after every use as opposed to only 9.5% in our study [17]. But this is higher than a study conducted in a UK medical school, where 22.4% of respondents had never cleaned their stethoscope and only 3.9% reported cleaning their stethoscopes after every patient, which is the required protocol [18].

In a research conducted in the department of pediatrics, The Medical City Ortigas Avenue, Pasig City, 34% of the respondents cleaned their stethoscopes more than once daily and only 33% had cleaned it within the past 24 hours, but the disinfection rate of stethoscopes came out to be lower in the hospitals we surveyed. Towering workload and deficient awareness were cited as reasons for not adhering to stethoscope cleanliness guidelines in the former study [19]. This also explains the fact that when the doctors and nursing staff of a hospital in Nigeria were surveyed to check for the cleanliness of stethoscopes, deplorable facts were obtained with the disinfection rate very low, and 90% doctors and nurses even claiming that they never cleaned their hands before or after using a stethoscope. They also expressed their desire for more seminars and guidance on the matter, to spread awareness about its importance [20].

The most popular cleaning agent reported in our study was alcohol wipes, with a usage rate of almost 52.3%. Cleaning with a wet or dry cloth or tissue paper is inadequate for bacterial control [21]. Only 4.5% of our respondents reported using a wet cloth for cleaning their stethoscopes. Our results have a higher percentage of alcohol use as compared to a study conducted in a hospital in Ujjain, where only 27.5% of the participants reported using ethyl alcohol [21].

The limitation of our study was that we were not able to enroll a large number of specialists. Moreover, the findings were self-reported so an information bias is possible.

A study was done in the UK, where swabs were collected from stethoscope diaphragms before and after a rigorous campaign for stethoscope hygiene. Swabs were collected one week prior to and one week after the application of strict protocols and advice to the hospital staff to maintain the cleanliness of stethoscopes. The number of colony-forming units dropped significantly from a median of 20 (range 0-50) in the first week to a median of eight (range 0-30). This showed that increased awareness of the problem and effective implementation of strict protocols and guidelines helped reduce the risk of infection and resulted in increased frequency of stethoscopes sterilization [22]. Unfortunately, the tertiary care hospitals that we surveyed had a very low number of people claiming such protocols existed and even then accountability measures were found to be non-existent.

The authorities should aim for providing education to healthcare professionals about the cleanliness of stethoscopes. Hospitals and clinics should issue proper written and visual guidelines. These protocols should be communicated at every level to ensure maximum compliance. Alcohol wipes should be made available in clinical settings for this purpose. Strict accountability measures should be taken by the hospital administration to access the cleanliness of the stethoscopes of healthcare professionals.

## Conclusions

Our study aimed to determine awareness among healthcare professionals regarding stethoscopes as a source of nosocomial infections. Participants included doctors, residents, final-year medical students, and nursing staff. The results of our study reflect awareness among healthcare professionals, as most of them acknowledged stethoscopes as a source. We also inquired about the frequency of the cleaning of stethoscopes by healthcare professionals and about the agent that was used for cleaning the stethoscope. In spite of the awareness of nosocomial infections, very few healthcare professionals actually regularly disinfect their stethoscopes. Only a small number of healthcare professionals cleaned their stethoscope per patient and most of them cleaned it using alcohol wipes. The majority of the participants were aware of stethoscopes being a source of nosocomial infections and believed in cleaning stethoscopes regularly. The majority of the participants also believed that their hospital did not issue any protocols for the decontamination of stethoscopes and accountability in regards to this was found to be non-existent. Further research can expand our recommendations. Doing this will not only be a noble service to society but also control the spread of infections within hospitals.
